# Genomic diversity and topical antimicrobial resistance in *Staphylococcus aureus* from clinical and carriage populations in the New York–New Jersey region

**DOI:** 10.1128/aac.00654-26

**Published:** 2026-06-30

**Authors:** Austin J. Terlecky, Klara V. Thom, Veronica W. Kan, Jianping Jiang, Elena Sashkina, Kelly K. Yen, Jose R. Mediavilla, Ogie Umasabor-Bubu, Malorie Rigor, Rina Melodie, Stefan H. F. Hagmann, Cristina Cicogna, Meghan W. Starolis, Julia A. Larsen, Akinyosoye Olufunmi, Albert Rojtman, Liang Chen, Barry N. Kreiswirth

**Affiliations:** 1 Hackensack Meridian Health Center for Discovery & Innovation, Nutley, New Jersey, USA; 2 Hackensack University Medical Center, Hackensack, New Jersey, USA; 3 Joseph M. Sanzari Children’s Hospital, Hackensack Meridian Health, Hackensack, New Jersey, USA; 4 Quest Diagnostics 7172 , Secaucus, New Jersey, USA; 5 Jersey Shore University Medical Center, Neptune Township, New Jersey, USA; 6 School of Pharmacy and Pharmaceutical Sciences, University at Buffalo 15497 , Buffalo, New York, USA; The Peter Doherty Institute for Infection and Immunity, Melbourne, Victoria, Australia

**Keywords:** genomic surveillance, mupirocin resistance, *Staphylococcus aureus*

## Abstract

*Staphylococcus aureus* remains a major public health concern due to its capacity for colonization, transmission, and antimicrobial resistance. Both methicillin-resistant (MRSA) and methicillin-susceptible *S. aureus* (MSSA) serve as reservoirs for resistance determinants, including those conferring reduced susceptibility to topical decolonization agents. We analyzed 2,330 *S. aureus* isolates collected between 2022 and 2025 from hospitals and community clinics in New Jersey and New York City, including 962 MRSA infection isolates and 1,368 nasal carriage isolates (310 MRSA and 1,058 MSSA) obtained from patients upon admission to intensive care, neonatal intensive care, and transplant units. Whole-genome sequencing and PCR characterized clonal lineages, plasmid structures, and resistance determinants, with focus on *mupA*, which mediates high-level mupirocin resistance. MRSA infection isolates were dominated by clonal complexes CC8 (63%), CC5 (21%), and CC30 (10%), whereas MSSA carriage isolates were more diverse, with CC398 most prevalent (15%). The *mupA* gene was detected in 26% of MRSA infection isolates, 50% of MRSA carriage isolates, and 36% of MSSA carriage isolates. Pairwise single-nucleotide polymorphism (SNP) analysis revealed most *mupA* variants differed by 0–2 SNPs across diverse lineages, consistent with recent horizontal dissemination. Long-read sequencing identified three major *mupA*-carrying plasmid groups—conjugative, mobilizable, and non-mobilizable—frequently co-harboring *qacC*, *aadD1*, *msr(A*), and *mph(C*). Non-mobilizable plasmids were confined to CC8, while mobilizable and conjugative variants circulated across multiple lineages. The high prevalence of *mupA*-bearing plasmids co-associated with quaternary ammonium resistance genes raises concerns regarding empiric mupirocin- and chlorhexidine-based decolonization strategies. Genomic surveillance and rapid resistance screening may be critical to preserving topical agent efficacy.

## INTRODUCTION

*Staphylococcus aureus* remains a major pathogen in healthcare and community settings, notable for its adaptability, asymptomatic colonization, resistance to multiple antibiotics, and ability to cause a diverse array of disease—from the common boil and wound infections to severe, life-threatening infections with metastatic complications and toxin-mediated syndromes. Both methicillin-resistant (MRSA) and methicillin-susceptible (MSSA) strains contribute significantly to this burden (1). While hospital-based infection control efforts have traditionally focused on nosocomial MRSA strains, there is increasing recognition that community and asymptomatic reservoirs of both MSSA and MRSA significantly contribute to the persistence and evolution of *S. aureus*, including the dissemination of antimicrobial resistance determinants (2).

Historically, MRSA was primarily confined to healthcare environments, emerging shortly after the clinical introduction of methicillin in the 1960s (3), and consistent with this observation, MRSA carriage in the community was rarely observed (4). However, the epidemiology of MRSA pivoted in the late 1990s as community-associated MRSA (CA-MRSA) clones such as USA300 spread widely outside hospital settings, infecting healthy individuals with no prior healthcare contact (5). The subsequent proliferation of these CA-MRSA clones diminished the previously distinct boundaries between healthcare-associated and community-associated lineages. Studies have since documented the bidirectional movement of clones between community and hospital reservoirs, facilitating the exchange of genetic material and resistance traits (6–9).

Colonization is a key reservoir for both MSSA and MRSA, with important implications for transmission dynamics and infection risk (10). While colonization prevalence varies globally, *S. aureus* carriage has been described at rates between 20% and 30%, with MRSA typically less than 10%, although potentially higher in low- to middle-income countries (11–13). Because colonization predisposes carriers to infection and facilitates transmission, reducing carriage has become a central focus of infection control strategies.

One of the cornerstone interventions for *S. aureus* control in healthcare settings is the use of topical antimicrobial agents, including mupirocin (Bactroban), fusidic acid (Fucidin), and chlorhexidine for decolonization, especially in high-risk environments such as intensive care units (ICUs), neonatal intensive care units (NICUs), and transplant wards. These agents are often used empirically to reduce colonization burden and prevent invasive disease in vulnerable patients.

Despite their widespread use, resistance to topical antimicrobials is not routinely assessed in clinical microbiology laboratories, and available surveillance data remain limited (14). Nonetheless, increasing reports document mupirocin resistance in both hospital and community isolates (14). High-level resistance is primarily mediated by the plasmid-borne *mupA* gene (*IleS2*), which encodes an alternative isoleucyl-tRNA synthetase that circumvents inhibition of the native enzyme. In contrast, low-level resistance usually results from point mutations in the chromosomal *ileS* gene that reduce mupirocin binding affinity. Importantly, *mupA*-carrying plasmids often harbor additional resistance determinants, thereby contributing to multidrug resistance phenotypes. Their prevalence varies across *S. aureus* lineages and epidemiological settings, with higher carriage rates observed in populations exposed to heavy empiric mupirocin use (15). For instance, genomic surveillance within a large U.S. healthcare system identified *mupA* in 22% of MRSA isolates, with strikingly high rates in the emerging ST8732 lineage of CC8 (95%). Notably, these plasmids frequently encoded *qac* genes, which confer resistance to quaternary ammonium compounds such as chlorhexidine (16).

To comprehensively assess topical antimicrobial resistance in *S. aureus* across healthcare and community settings, we analyzed a regionally diverse set of isolates capturing both clinical and carriage reservoirs: MRSA infections from acute care hospitals and outpatient clinics and MRSA/MSSA from nasal carriage and bloodstream infections in high-risk inpatient units. Whole-genome sequencing (WGS) was applied to all infection isolates to enable detailed lineage and plasmid characterization, while polymerase chain reaction (PCR) was used for rapid *mupA* and *spa* typing in colonization isolates. This approach allowed for high-resolution genomic analysis of resistance mechanisms in invasive disease and provided broader insights into prevalence and lineage trends among colonizing strains.

Our study provides a regionally focused, multi-setting analysis of topical antimicrobial resistance, lineage diversity, and plasmid characterization in *S. aureus*, incorporating genomic and molecular data from both infection and colonization contexts. This work improves the understanding of resistance dynamics across overlapping hospital and community reservoirs and highlights the importance of integrated surveillance approaches.

## MATERIALS AND METHODS

### Isolate collection

*S. aureus* isolates were collected from May 2022 to August 2025. The following were included in this collection. A total of 651 MRSA blood, wound, urine and respiratory infection isolates were collected from five New Jersey hospitals: Jersey Shore University Medical Center (JSUMC), Riverview Medical Center (RMC), Ocean University Medical Center (OUMC), Southern Ocean Medical Center (SOMC), and Bayshore Medical Center (BMC). These hospitals collectively serve a coastal catchment area of approximately 50 miles in New Jersey and provide comprehensive acute and specialty care, including emergency medical services. All isolates were processed at the JSUMC Microbiology Lab and sent to the Center for Discovery and Innovation (CDI) for genomic analysis. A total of 311 MRSA blood, wound, urine and respiratory infection isolates were also collected from community clinics and analyzed at the Quest Diagnostics laboratory in Clifton, NJ. These infections are associated with patients who live in communities with zip codes from northern New Jersey and New York City. Each of the 962 infection isolates represents a distinct patient. Unique patient representation was ensured at the point of collection and prior to de-identification.

In addition to clinical infection isolates, 5,663 nasal swabs collected for *S. aureus* colonization screening were obtained from patients admitted to the ICU, NICU, and transplant wards at Hackensack University Medical Center (HUMC). HUMC is a general acute care teaching and research hospital in northern New Jersey with approximately 800 beds, including 48 adult ICU beds and 15 pediatric ICU beds. From these samples, 1,368 unique *S. aureus*–positive cultures were identified, defined as one representative isolate per patient per admission screening encounter, while the remaining swabs were *S. aureus*–negative. When multiple *S. aureus* colonies were recovered from a single swab, a single representative colony was selected for further characterization. All 1,368 *S. aureus*–positive isolates were transferred to the CDI for molecular screening and genomic sequencing.

This study was exempt from Institutional Review Board approval because it involved de-identified bacterial isolates obtained during routine clinical care.

### Culturing and isolate storage

All *S. aureus–*positive isolates were plated on mannitol salt agar, which serves both as a selective and differential medium. The medium is composed of mannitol, salt, and pH indicator phenol red. Salt inhibits growth of most bacteria but selects for Staphylococci as a result of their high salt tolerance. *S. aureus* can ferment mannitol and produce acid that turns the media yellow in the presence of phenol red. After a 12–24 h incubation period, the isolates that grew were stocked in 1 mL of 20% LB glycerol and stored at −80°C.

### DNA isolation

To isolate DNA for *spa* typing and *mupA* PCR analysis, bacterial growth from the agar plate was resuspended in 100 µL of ddH₂O mixed with 1 µL of 2 mg/mL lysostaphin and boiled for 10 min. After pelleting at 4,500 RPM for 5 min, the supernatant was diluted 1:5 in ddH₂O, and the dilution was used as a template for PCR.

To prepare isolates for whole-genome sequencing (WGS), DNA extraction was performed using the Wizard Genomic DNA Purification Kit from Promega (Promega Corporation; Madison, WI, USA). Lysostaphin was used as the lytic enzyme for the initial pretreatment step to weaken the cell wall.

### PCR screening for mupA

After DNA was extracted through boiling, isolates underwent a PCR screening for the presence of *mupA*. Primers were designed based on conserved regions identified through WGS of *mupA-*positive *S. aureus* sequences. Primer sequences were as follows: forward primer (mupA436F): 5′-AGGAGGCTGAAAAGTTTTGACA-3′ and reverse primer (mupA436R): 5′-GGAGTCCATGTCAACCCAGT-3′. PCRs were performed using Thermo Scientific DreamTaq DNA Polymerase, following the manufacturer’s protocol.

### Spa typing

*Spa* types were determined via WGS or PCR followed by Sanger sequencing, as previously described (17). The repeat region of *S. aureus* protein A gene was amplified with forward primer (TIGR_F): 5′-GCCAAAGCGCTAACCTTTTA-3′ and reverse primer (TIGR_R): 5′-TCCAGCTAATAACGCTGCAC-3′. The primer set amplified a fragment of 400–700 bp, depending on the number of repeats present in each individual isolate. PCRs were performed according to the Thermo Scientific DreamTaq DNA Polymerase protocol. Generated PCR fragments were Sanger-sequenced, and chromatograms with base calls were analyzed using the spaTyper tool (18).

### Whole-genome sequencing and genomic analysis

After whole-genome DNA was prepared as described above, isolates underwent short-read WGS using the Illumina HiSeq 2500 platform with 2 × 150 bp paired-end reads (Illumina, Inc; San Diego, CA, USA). Isolates undergoing long-read sequencing were sequenced with the Oxford Nanopore GridION platform (Oxford Nanopore Technologies plc; Oxford, UK). Illumina sequences were trimmed using Trimmomatic v0.39 (19) and *de novo-*assembled using SPAdes v4.0.0 (20). Those isolates sequenced with both Illumina and Nanopore were hybrid-assembled using Unicycler v0.5.1 (21), enabling resolution of complete chromosome and plasmid sequences. Isolates sequenced only through the Nanopore platform were assembled using Flye v2.9.6 (22). The presence of *mupA* was identified using AMRFinderPlus v4.0.23 (23) on the assembled genomes.

All sequencing data generated in this study have been deposited to the NCBI Sequence Read Archive under BioProject accession PRJNA549322. Sequencing and assembly quality metrics including sequencing platform, total reads, genome size, and estimated genome coverage for all sequenced isolates are provided in Table S1. Isolate-level metadata, including the year of collection, collection site, and infection versus carriage designation, are provided in Table S2.

Lineage assignments were performed using the MLST command line tool (24, 25) and *spa* typing, with clonal complexes inferred from *spa* types. *Spa* typing targets the polymorphic variable number tandem repeat (VNTR) region within the Staphylococcal protein A (*spa*) gene (17). The amplified fragment is sequenced using Sanger sequencing (as described above in *spa* Typing), and the resulting repeat pattern is used to assign a specific *spa* type. These *spa* types can be used to infer clonal complexes, as described by Mediavilla et al. (26).

Assembled plasmids were characterized by antimicrobial resistance content with AMRFinderPlus (23), while Plasmidfinder (27) was used to identify plasmid replicons, and MOB-typer v3.1.9 (28) was used to characterize predicted mobility, identifying plasmids that are conjugative, mobilizable, or non-mobilizable. Visual plasmid comparisons were compiled using EasyFig (29).

### Minimum-inhibitory concentration (MIC) testing

Susceptibility testing included mupirocin broth microdilution MIC assays in accordance with Clinical and Laboratory Standards Institute (CLSI) guidelines (30). Cultures were grown in cation-adjusted Mueller-Hinton (MH) broth and subsequently adjusted to an optical density at 600 nm (OD₆₀₀) of 0.2. A microtiter plate containing MH broth and serial twofold dilutions of appropriate antibiotics were inoculated with 5 × 10⁵ colony-forming unit (CFU) bacterial culture and grown overnight at 37°C. The lowest concentration without any visible growth was interpreted as the MIC. Interpretive thresholds were applied based on previously published criteria: strains with mupirocin MICs of ≤4 µg/mL were considered susceptible; MICs between 8 and 256 µg/mL indicated low-level resistance; and MICs of ≥256 µg/mL indicated high-level resistance (14).

## RESULTS

### Lineage diversity

**Fig 1:**
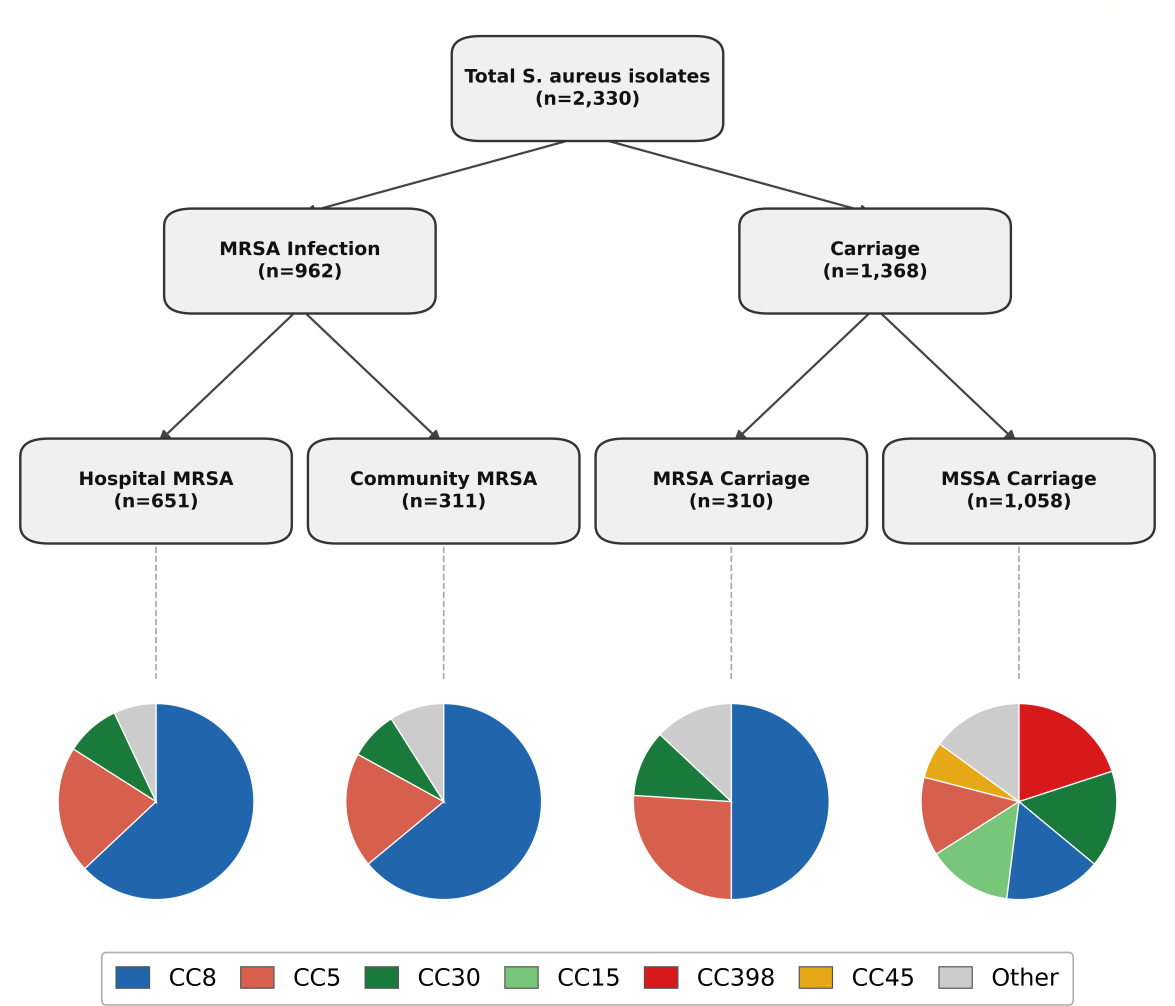
Flow chart and clonal complex distribution of the *S. aureus* collection. Flow chart depicting the structure of the *S. aureus* isolate collection. Isolates were divided into infection and carriage groups. MRSA infections were further separated into isolates obtained from hospitals and those from the community, while carriage isolates were split into MRSA and MSSA. For each subcollection, the distribution of clonal complexes (CCs) is shown as pie charts.

A total of 2,330 *S. aureus* isolates were included in this study, encompassing both clinical infections and nasal carriage. Among these, 962 isolates (41%) were MRSA infection isolates, defined as isolates recovered from clinical specimens (blood, urine, respiratory, or wound), obtained from five acute care hospitals (*n* = 651) and from community populations (*n* = 311). The remaining 1,368 isolates (59%) were cultured from 5,663 nasal swabs obtained in ICU patients, comprising 310 MRSA and 1,058 MSSA, indicating a total carriage rate of 24% (5.5% MRSA and 18.7% MSSA). Figure 1 illustrates the distribution of infection and carriage isolates, stratified by MRSA and MSSA status.

Among the 962 MRSA infecting isolates, clonal complex 8 (CC8) was the predominant clonal group, representing 63% of these clinical isolates, followed by CC5 (21%) and CC30 (10%). Clonal complexes CC45, CC1, CC22, CC15, and CC121 define the remaining 6% of the isolates. This distribution of the infecting hospital isolates paralleled the results from community populations, with CC8, CC5, and CC30 making up 64%, 19%, and 8%, respectively.

Among the MRSA nasal carriage isolates collected, CC8 was still the most predominant clone at 42%, with CC5 next at 22%, followed by CC30 at 9%. The remaining 27% of isolates were made up of CC1, CC15, CC398, CC97, CC121, CC72, CC22, CC59, CC80, and CC88.

The 1,058 MSSA carriage isolates were markedly more diverse. CC398 was the most frequent clone, making up 15% of colonizing isolates. This was followed by CC30 and CC8 (12% each), CC15 (11%), and CC5 (10%). CC45 accounted for an additional 5%. The remaining 35% were made up of CC1, CC22, CC121, CC97, CC72, CC20, CC152, CC25, CC80, and CC88.

### Mupirocin resistance genotyping

**Fig 2:**
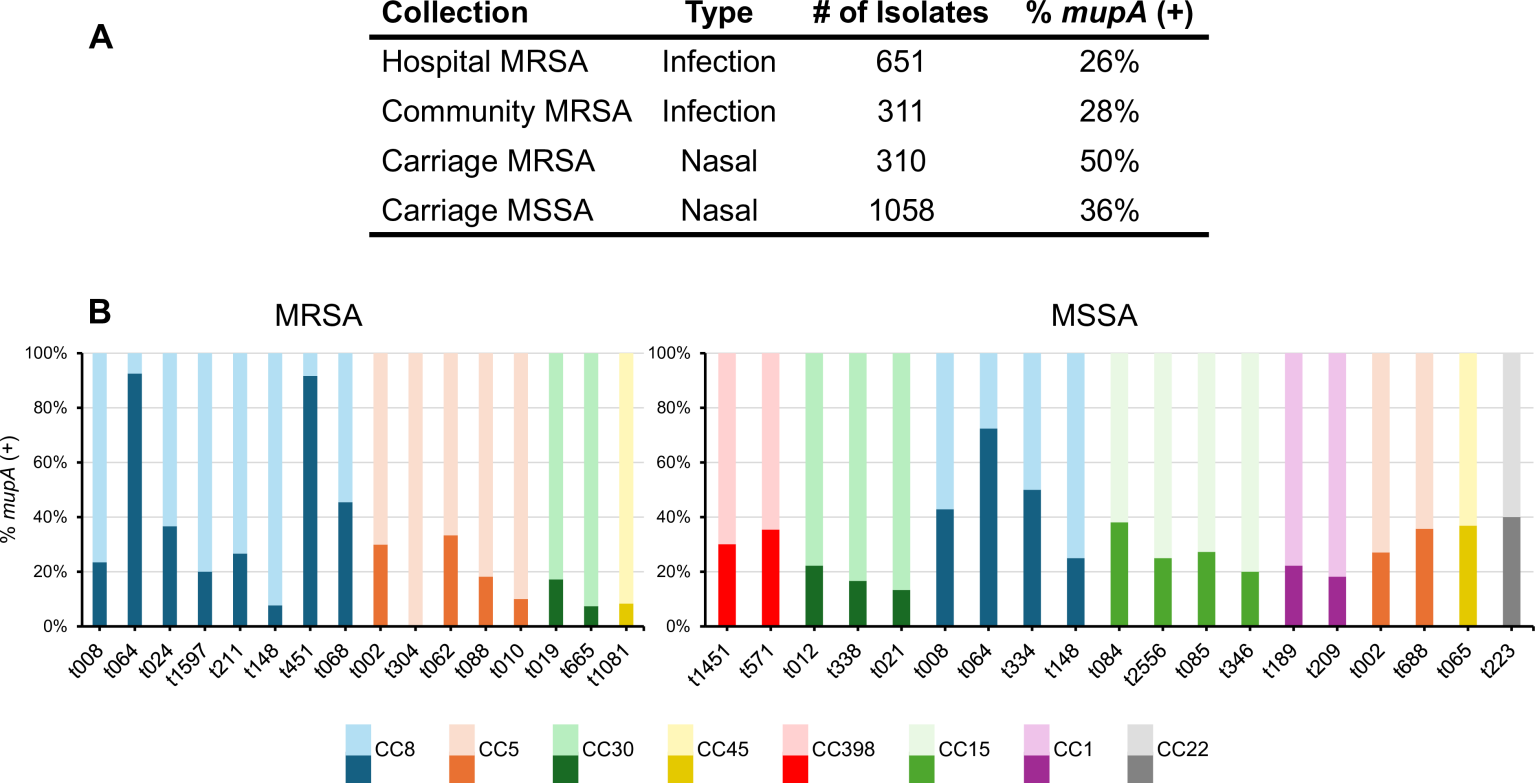
Distribution of predominant *spa* types and *mupA* presence across *S. aureus* subcollections. (**A**) Table showing the number of isolates in each subcollection and the corresponding percentage carrying *mupA*. (**B**) Stacked bar charts of the most frequent *spa* types (more than 10 isolates collected) within MRSA and MSSA, representing 72% of all MRSA isolates and 54% of all MSSA isolates, respectively. Each bar represents all isolates of a given *spa* type (100%), subdivided to indicate the proportion of *mupA*-positive and *mupA*-negative isolates. Bar colors denote the clonal complex associated with each spa type and are ordered by most common CC before the most common *spa* type.

High-level mupirocin resistance was genotypically inferred based on detection of intact *mupA* across the full collection. Phenotypic confirmation by broth microdilution MIC testing was performed on the subset of 56 isolates that underwent Nanopore sequencing, as described below. The *mupA* gene was detected in MRSA infections from both hospital and community sources, as well as in MSSA and MRSA nasal carriage isolates. The prevalence of *mupA* across these populations is summarized in Fig. 2.

Among the 962 MRSA infecting isolates, *mupA* was present in 26%, with nearly identical rates in the hospital (26%) and community (28%) collections. In contrast, carriage isolates showed a substantially higher prevalence, with *mupA* detected in 50% of MRSA and 36% of MSSA. *mupA* was distributed across diverse lineages of both MRSA and MSSA, with rates ranging from 20% to 40% among the most common clones: CC8, CC5, and CC30. Notably, certain spa types within CC8 exhibited elevated frequencies. Among CC8 MRSA spa types t064 (*N* = 131), t451 (*N* = 12), and t723 (*N* = 6), the *mupA* gene was identified in 92%, 92%, and 100%, respectively. The strong association of mupirocin resistance among t064 MRSA was also observed among the 28 t064 MSSA carriage isolates as 75% harbored *mupA*.

To assess *mupA* sequence conservation across this collection, we extracted *mupA* coding sequences from all Illumina draft genomes (*N* = 234), aligned them using MAFFT, and computed pairwise SNP distances using snp-dists. The *mupA* gene was remarkably conserved as most sequences differed by 0–2 SNPs across diverse clonal backgrounds including CC8, CC5, and CC30. A small subset of isolates harbored more divergent *mupA* variants, with pairwise distances reaching up to nine SNPs from the dominant cluster; these divergent sequences were identified predominantly among non-CC8 lineages and showed no difference in phenotypic resistance determined by MIC testing.

### Plasmid characterization

To characterize the plasmids, particularly those harboring *mupA*, a subset underwent long-read sequencing with Nanopore, facilitating their assembly. The selected isolates represented diverse clonal complexes and spa types of both MRSA and MSSA. A total of 56 *mupA* plasmids were assembled from these positive isolates.

**Fig 3:**
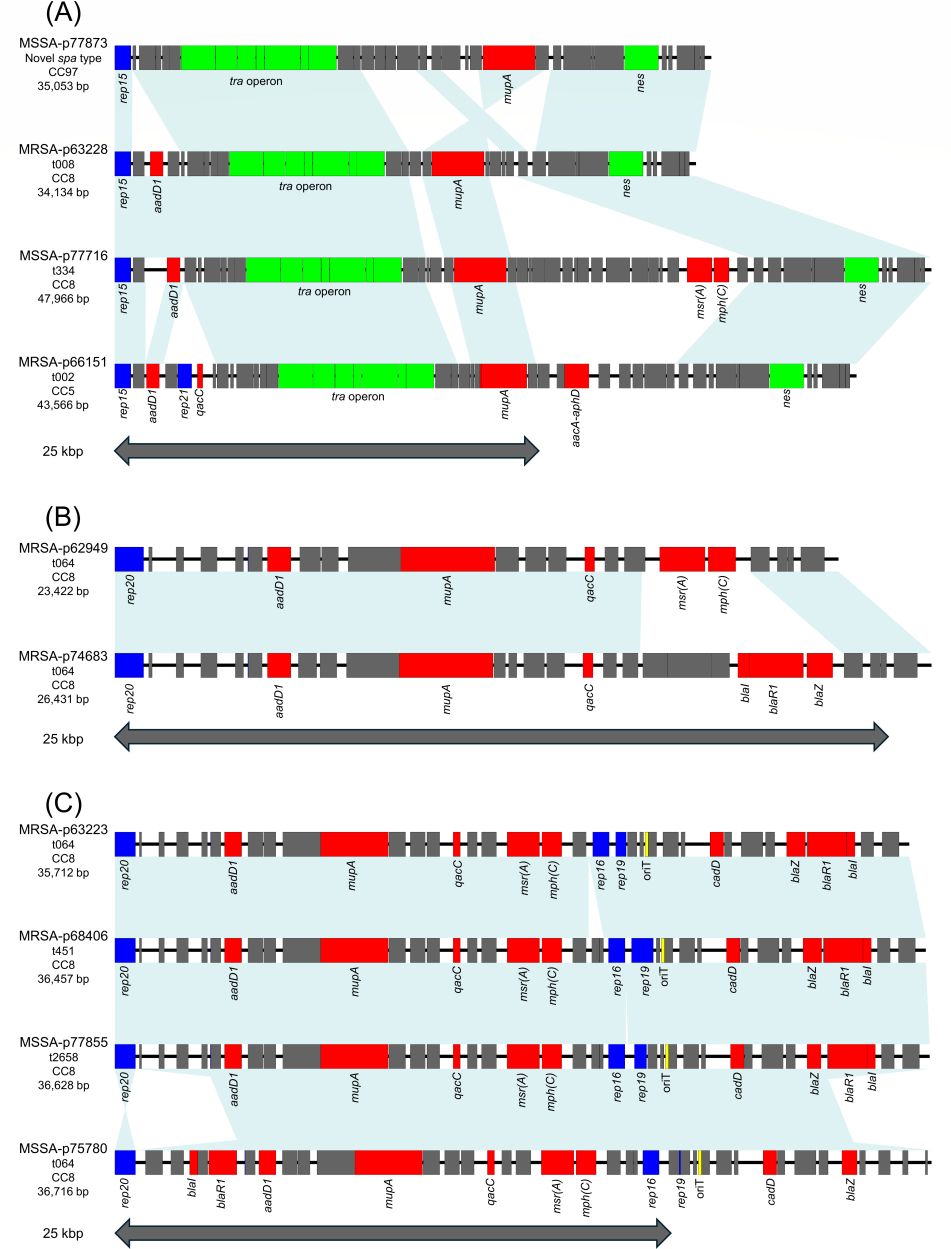
Alignment and grouping of *mupA-*positive plasmids. (**A**) Subset of conjugative plasmids defined by the presence of a *tra* operon and relaxase gene (*NES*), representing multiple clonal complexes and *spa* types of both MRSA and MSSA. (**B**) Subset of non-mobilizable plasmids lacking a *tra* operon, relaxase, and *oriT*. This group is composed exclusively of MRSA isolates of *spa* type t064. (**C**) Subset of mobilizable plasmids defined by the presence of an origin of transfer (*oriT*) but lacking conjugation machinery. This group also includes isolates from multiple clonal complexes and *spa* types of MRSA and MSSA. Antimicrobial resistance genes, including *mupA*, are indicated in red; replicon origins in blue; conjugation-associated genes in green; and origins of transfer in yellow. One isolate without a defined *spa* type in group A is labeled as “novel *spa* type,” it carries a previously unreported repeat pattern (UEFMBBBPB or 07-13-21-17-34-34-34-33-34 in Kreiswirth or Ridom nomenclature, respectively).

The *mupA-*positive plasmids were categorized into three groups based on predicted mobility and genetic architecture (Fig. 3). Group A plasmids were conjugative, defined by the presence of a *tra* operon and a relaxase gene (*nes*). Although relatively uncommon, these plasmids were identified across multiple spa types of both MRSA and MSSA. All Group A plasmids carried the rep15 replicon, and they frequently encoded additional resistance genes, including *aadD1*, *msr(A*), *mph(C*), and *qacC*. Group C plasmids were predicted to be mobilizable, containing an origin of transfer (*oriT*) but lacking conjugation genes. This group was also distributed across diverse genetic backgrounds of both MRSA and MSSA and consistently carried *mupA* together with *qacC*, *aadD1*, *msr(A*), and *mph(C*). A conserved resistance cassette comprising *cadD*, *blaZ*, *blaR1*, and *blaI* was also present. Replicons identified within this group included rep20, rep16, and rep19. Group B plasmids were predicted to be non-mobilizable, lacking both conjugation-associated genes and an *oriT*. Despite the absence of transfer-associated elements, their overall organization closely resembled that of Group C plasmids, corresponding to the region containing rep20, *mupA*, and associated resistance determinants, but lacking the rep19–rep16 segment harboring the *oriT* in Group C. Of note, the plasmids in group B were confined exclusively to MRSA isolates of *spa* type t064.

To examine the association between plasmid mobility groups and clonal complexes, replicon types and mobility predictions from MOB-typer were cross-referenced with CC assignments across the 56 long-read sequenced isolates. Non-mobilizable Group B plasmids were restricted almost entirely to CC8, accounting for all 22 non-mobilizable plasmids in the data set. Mobilizable Group C plasmids were distributed across CC8 (*n* = 13), CC5 (*n* = 6), CC398 (*n* = 1), and one isolate of undetermined clonal complex. Conjugative Group A plasmids were identified across CC8 and CC5, with one additional isolate of undetermined clonal complex.

This characterization confirmed that *mupA* plasmids were not confined to a single genetic background but circulated across both MRSA and MSSA within multiple clonal complexes, including CC8, CC5, CC30, CC15, CC45, CC398, and CC1.

### Minimum-inhibitory concentration

MIC testing was performed on all 56 *mupA-*positive isolates that underwent Nanopore sequencing. Four isolates harbored a frameshift mutation (Ile94fs) in *mupA*, genotypically predicting loss of resistance; all four were phenotypically susceptible (MIC ≤0.5 µg/mL). These comprised three CC8, *spa* type t064 isolates (two MRSA infection isolates and one MSSA nasal carriage isolate) and one CC5, *spa* type t002 MRSA infection isolate. Among the 52 isolates with intact *mupA*, 50 (96%) displayed high-level mupirocin resistance with MICs ≥ 256 µg/mL. The remaining two isolates, both CC8, had MICs of 64 and 128 µg/mL.

## DISCUSSION

Across our regional cohort, *S. aureus* carriage was detected in 24% of individuals, with MSSA accounting for 18.7% and MRSA for 5.5%. These estimates align closely with those of previous studies from urban U.S. populations and international meta-analyses, which report carriage rates between 25% and 35% (4, 11). In a 2008 study of 256 healthcare providers in an emergency room setting including EMT personnel, *S. aureus* carriage ranged between 38.5% and 57.7%, with MRSA comprising 15.2% of carriers (31). Earlier national data from the 2001–2004 NHANES surveys reported *S. aureus* carriage of 28%–32% and MRSA carriage between 0.8% and 1.5% among noninstitutionalized individuals (32). While the NHANES data were collected in the early 2000s, they remain the most comprehensive nationally representative estimates of *S. aureus* carriage available for the U.S. population. The higher MRSA proportion we observed here likely reflects our hospital population, which includes patients from high-risk wards.

While MRSA continues to be prioritized due to its clinical severity and multidrug resistance, the predominance of MSSA in asymptomatic colonization suggests that methicillin susceptibility does not equate to reduced clinical significance. MSSA remains a major cause of invasive infections and demonstrates considerable genetic diversity and virulence potential, emphasizing its ongoing importance in both transmission and disease burden (33, 34).

Both MRSA carriage and infection populations in our study revealed limited overall diversity. CC8 was the predominant genetic background in both clinical and carriage isolates, followed by CC5 and CC30. This population structure mirrors established U.S. epidemiologic patterns and furthers the observations that CC8 isolates predominate in both settings (32, 35). The shift in MRSA epidemiology in the United States—marked by the rise and dominance of clonal complex 8 (CC8)—was clearly demonstrated in two surveillance studies conducted by the CDC. In the first setting, Tenover et al. (32) analyzed MRSA nasal carriage from 2001 to 2004 and reported that USA100 (CC5, spa type t002) was the predominant genetic background, consistent with its predominance as a hospital pathogen. However, the study also showed the emergence of USA300 (CC8, spa type t008), foreshadowing the changing epidemiology (32). A second surveillance study conducted between 2009 and 2010 evaluated MRSA isolates from 23 U.S. hospitals and highlighted the successful emergence of spa type t008 (CC5/USA300) and its co-dominance with spa type t002 (CC5/USA100) as together, they accounted for nearly all carriage and bloodstream isolates (35).

Our results demonstrate the continuation of that trend, with CC8 now firmly established as the leading MRSA lineage in both infection and carriage across our region. The presence of CC8, CC5, and CC30 in both clinical and colonizing populations suggests that these lineages possess broad ecological fitness, capable of persisting across hospital and community environments. Our findings reinforce this national picture, particularly that MRSA remains a highly clonal population defined by these two dominant lineages, while also suggesting an ongoing expansion of CC8 relative to CC5. Of note, we also observed an increased representation of spa type t064. Historically comprising roughly 3%–5% of MRSA isolates (32, 35), t064 accounted for approximately 10%–15% of our MRSA isolates, depending on infection or carriage source, suggesting a modest but notable rise of this lineage within our region. Among MRSA carriage isolates, CC8 was the predominant lineage (42%), followed by CC5 (22%) and CC30 (9%).

In contrast to the restricted diversity observed in MRSA, MSSA carriage isolates in our cohort were markedly diverse. Multiple clonal complexes were represented, but CC398 emerged as the most frequent (15%), followed by CC30 and CC8 (12% each), CC15 (11%), and CC5 (10%). The high diversity of MSSA lineages aligns with prior findings from both U.S. and European studies, which have consistently shown that methicillin-susceptible populations are far more genetically variable compared to MRSA (32, 36).

In the nationwide NHANES analysis, Tenover et al. (32) likewise reported that MSSA carriage was genetically diverse, spanning multiple PFGE-defined lineages such as USA200, USA600, USA900, and USA800. Notably, CC398 was not detected among U.S. carriage isolates from 2001 to 2004. A later U.S. study focused on MSSA infections between 2004 and 2010 (33) also revealed extensive spa type diversity (274 unique spa types across 708 isolates) but did not identify CC398 among bloodstream or skin/soft-tissue infections (33). Instead, the most common MSSA lineages were spa type t002 (CC5) and spa type t008 (CC8), the same genetic backgrounds associated with epidemic MRSA clones. Similarly, Monecke et al. (36), who characterized MSSA from asymptomatic carriers in Germany between 2005 and 2008, observed broad lineage diversity—including CC8, CC15, CC30, and CC45—but again found no evidence of CC398 in human carriage. In contrast, our data set provides evidence that CC398, originally recognized as a livestock-associated MRSA lineage, is now an established human nasal carriage within the U.S. population.

The emergence of CC398 in human MSSA carriage parallels findings from Europe and the U.S., where human-adapted variants of MSSA-CC398 have increasingly been reported (37, 38). Originally identified as livestock-associated MRSA in European pig farms (39), this lineage has since evolved into a human-adapted form, acquiring immune evasion clusters (IEC), altered adhesion profiles, and enhanced biofilm capacity that facilitate colonization and transmission in human hosts (40). The high prevalence of CC398 is notable, given its recent emergence as a globally relevant MSSA clone subsequent to its identification as a livestock-associated MRSA clone in the early 2000s (37, 39, 41). Of clinical concern, MSSA-CC398 has become an increasingly common cause of bloodstream and orthopedic infections, including in individuals without occupational exposure to animals (34, 37). These findings align with previous regional and international reports describing the establishment and persistence of MSSA-CC398 in urban healthcare and community settings (37, 38, 40–42).

A central finding of our study is the high prevalence and broad genomic distribution of *mupA*-mediated mupirocin resistance across the NYC/NJ region. The *mupA* gene was detected across diverse genetic backgrounds spanning CC8, CC5, CC30, and MSSA lineages and was associated with plasmid backbones representing a range of transfer potential, underscoring the breadth of its regional distribution across both infection and carriage populations. Detected in up to 50% of MRSA carriage isolates and present in MSSA (36%), *mupA* was far more widespread than previously recognized, and its spread to diverse genetic backgrounds parallels previous studies (14, 43). Importantly, our data reinforce that topical antimicrobial resistance is not confined to traditional hospital-associated MRSA but is embedded across multiple lineages and settings, including MSSA, necessitating a reevaluation of empirical-based decolonization strategies. Recent surveillance from a large U.S. healthcare system similarly reported *mupA* in 22% of MRSA isolates, with particularly high prevalence in the emergent ST8732 (ST8 single-locus variant) lineage (95%) and frequent co-carriage with *qac* genes that mediate reduced chlorhexidine susceptibility (16). Together with our data, these findings suggest that mupirocin and biocide resistance are becoming increasingly entrenched across both dominant and novel lineages, amplifying concerns about decolonization efficacy.

The conservation of *mupA* sequences across this collection further informs our understanding of its spread. Pairwise SNP distances revealed that the majority of *mupA* variants differed by 0–2 SNPs from one another irrespective of the clonal background, with this near-identity maintained across CC8, CC5, and CC30. A small subset of more divergent sequences, differing by up to nine pairwise SNPs from the dominant cluster, was observed predominantly among non-CC8 lineages. This pattern, combined with the plasmid distribution data described below, is consistent with *mupA* spreading primarily by horizontal transfer across lineages, with clonal expansion contributing to its high prevalence in certain genetic backgrounds, most notably CC8 *spa* type t064.

Long-read sequencing demonstrated that *mupA* was distributed across several plasmid backbones with distinct mobility profiles. Conjugative Group A plasmids, although infrequent, were detected in both MRSA and MSSA and carried rep15 alongside multiple resistance determinants. More commonly, *mupA* was associated with mobilizable Group C plasmids, which shared a conserved resistance cassette and occurred across diverse genetic backgrounds. Non-mobilizable Group B plasmids represented a truncated variant of this architecture and were restricted to MRSA *spa* type t064.

To contextualize the plasmid backbones identified in this collection, each assembled backbone was searched against publicly deposited *Staphylococcus* sequences in NCBI (≥90% identity, ≥5,000 bp, ≥50% query coverage). The mobilizable Group C backbone was the most narrowly distributed despite its prevalence in our collection, returning 353 hits restricted exclusively to *S. aureus* at 97.8%–100% nucleotide identity. The non-mobilizable Group B backbone similarly returned hits restricted to *S. aureus* (*n* = 11), at 98.5%–100% identity, consistent with a recently derived, host-adapted variant. The conjugative Group A backbone was the most broadly distributed, returning 362 hits across 14 organisms at 90.6%–100% identity. These included both *S. aureus* and other staphylococcal species such as *S. pseudintermedius*, *S. epidermidis*, *S. capitis*, and *S. hominis*, reflecting the broad host range associated with conjugative elements.

The dominance of *mupA*-positive spa type t064 isolates suggests that this lineage has likely undergone clonal expansion driven by the stable maintenance of Group B plasmids. The confinement of these non-mobilizable plasmids to *spa* type t064, coupled with their structural similarity to the mobilizable Group C plasmids, indicates that horizontal transfer may have seeded the initial acquisition of *mupA*, after which clonal proliferation sustained its high prevalence. Together, these findings highlight that *mupA* resides on a structurally diverse but genetically related plasmid family that varies primarily in transfer potential. The frequent co-location of *qac* and other antimicrobial resistance genes within these plasmids underscores the potential for co-selection and persistence even in the absence of mupirocin exposure. While conjugative elements were uncommon, the coexistence of mobilizable and non-mobilizable variants across multiple *S. aureus* lineages, including the apparent clonal expansion within *spa* t064, illustrates the combined roles of horizontal and vertical gene transfer in maintaining topical antimicrobial resistance.

We confirmed with HUMC infection prevention personnel that the decolonization protocol during the study period included universal chlorhexidine gluconate bathing for patients in the adult ICU and stem cell transplant ward, with intranasal mupirocin administered only to a significant minority of patients meeting specific clinical criteria on admission. These protocols are noted here as the context for interpreting the mupirocin-resistant carriage prevalence data.

Taken together, these findings raise critical concerns about the sustainability of current empiric decolonization protocols. In high-risk hospital units such as ICUs, NICUs, and transplant wards, the routine use of mupirocin and chlorhexidine without prior resistance testing may inadvertently select for resistant strains. Our observation of high *mupA* prevalence in lineages already dominant in infection (e.g., CC8 and CC5) suggests that empiric strategies may contribute to prolonged colonization and recurrent infection. This aligns with recent reports showing that the combined carriage of *mupA* and *qac* genes in MRSA correlates with potential decolonization failure, highlighting the urgency of integrating resistance screening into infection prevention workflows (16). As resistance becomes increasingly embedded in the community and hospital reservoirs, decolonization strategies must transition from empirical to guided models. Routine PCR-based resistance screening for genes like *mupA*, *fusB/C*, and *qacA/B* could enable real-time tailoring of interventions. These tools, given their scalability and rapid turnaround time, are particularly well suited for integration into diagnostic workflows in high-burden environments. Our findings support the broader implementation of such approaches.

Our study builds on prior genomic surveillance efforts that document lineage diversity and resistance profiles among both infecting and colonizing *S. aureus* isolates (32, 35, 44). These findings underscore the value of regional surveillance in capturing localized trends in resistance gene distribution, clonal emergence, and plasmid content. By integrating epidemiological, genomic, and phenotypic data, our analysis of different strain populations offers a comprehensive regional snapshot of topical antimicrobial resistance and its distribution in regard to plasmid structure, genetic backgrounds, and spread between hospital, community, and carriage reservoirs. With the understanding that routine phenotypic testing of mupirocin resistance is not performed, our findings carry direct implications for infection control strategies and decolonization practices and highlight the need to evaluate the extent of resistance in both infecting and carriage *S. aureus* isolates before topical treatment is implemented.

The strengths of this study lie in its scale and methodological breadth. By analyzing over 2,300 isolates from diverse clinical and epidemiological settings and applying both genomic and molecular tools, we were able to resolve clonal structure, resistance gene content, and plasmid diversity at high resolution. The inclusion of both MRSA and MSSA isolates enhances our understanding of resistance ecology and reflects the complex reality of *S. aureus* transmission in hospitals and the community.

However, some limitations should be noted. First, phenotypic susceptibility testing was not uniformly performed, limiting our assessment of low-level resistance and phenotypic-genotypic concordance. Second, PCR-based screening of colonization isolates did not fully resolve the genomic context of resistance genes, particularly in cases involving plasmid-mediated elements. Third, we lacked patient-level metadata (e.g., prior mupirocin exposure and clinical outcomes), which constrained our ability to evaluate risk factors or assess the clinical impact of resistance carriage. Future studies that incorporate these dimensions will be essential to translate genomic findings into infection control strategies.

### Conclusion

In summary, the genomic epidemiology of topical antimicrobial resistance in *S. aureus* is dynamic and embedded in both community and healthcare ecosystems. Addressing it will require coordinated efforts in diagnostic stewardship, real-time surveillance integration, and a shift from empiric to more guided decolonization strategies. Our findings contribute to a growing body of evidence that resistance to topical antimicrobials in *S. aureus* is a widespread and evolving phenomenon shaped by reservoirs spanning hospitals and communities. Importantly, surveillance must expand beyond MRSA to include MSSA, which continues to dominate colonization in multiple cohorts.

## SUPPLEMENTAL MATERIAL

Supplemental material aac.00654-26-s0001.docx Supplemental table legends.

Table S1 aac.00654-26-s0002.xlsx Sequencing and genome assembly quality metrics.

Table S2 aac.00654-26-s0003.xlsx Metadata for all isolates.

ASM does not own the copyrights to Supplemental Material that may be linked to, or accessed through, an article. The authors have granted ASM a non-exclusive, world-wide license to publish the Supplemental Material files. Please contact the corresponding author directly for reuse.
