# Supplementary material for: Genomic diversity and topical antimicrobial resistance in Staphylococcus aureus from clinical and carriage populations in the New York–New Jersey region
Source: Antimicrob Agents Chemother. 2026 Jun 30;70(8):e00654-26. doi: 10.1128/aac.00654-26 (PMC13436320; doi:10.1128/aac.00654-26)
Supplement: Supplementary file 1 [file aac.00654-26-s0001.docx]

**Supplemental Table Legends**

**Supplemental Table S1. Sequencing and genome assembly quality metrics.**

Table containing quality metrics for genome sequencing and assembly of all isolates contained in this study. 962 isolates were sequenced using Illumina, 36 of these isolates were also sequenced using Nanopore. An additional 20 isolates were sequenced only by Nanopore. These sequencing experiments are deposited to the NCBI Sequence Read Archive under BioProject Accession: PRJNA549322

**Supplemental Table S2. Metadata data for all isolates**

Table containing metadata information for all 2330 isolates contained in this study.
